# TMEFF2 promoter hypermethylation is an unfavorable prognostic marker in gliomas

**DOI:** 10.1186/s12935-021-01818-x

**Published:** 2021-03-04

**Authors:** Sidi Xie, Yunxiao Zhang, Tao Peng, Jinglin Guo, Yongfu Cao, Jing Guo, Xiaofeng Shi, Yaqin Li, Yawei Liu, Songtao Qi, Hai Wang

**Affiliations:** 1 Department of Neurosurgery, Nanfang Glioma Center, Nanfang Hospital, Southern Medical University, Guangzhou North Road, Guangzhou, 510515 Guangdong People’s Republic of China; 2 Laboratory for Precision Neurosurgery, Nanfang Hospital, Southern Medical University, Guangzhou, 510515 Guangdong People’s Republic of China; 3 Department of Neurosurgery, First Hospital of Jilin University, Changchun, 130021 Jilin People’s Republic of China; 4 Department of Neurosurgery, The Fifth Affiliated Hospital of Guangzhou Medical University, Guangzhou, 510000 Guangdong People’s Republic of China; 5 Epilepsy Center, Guangdong Sanjiu Brain Hospital, Guangzhou, 510000 Guangdong People’s Republic of China; 6 Department of Neurosurgery, Longgang Central Hospital of Shenzhen, Shenzhen, 518116 Guangdong People’s Republic of China; 7 The Seventh Affiliated Hospital, Sun Yat-sen University, Shenzhen, 518107 Guangdong People’s Republic of China

**Keywords:** TMEFF2, Glioma, DNA methylation, IDH1, Prognosis

## Abstract

**Background:**

Transmembrane protein with EGF-like and two follistatin-like domains 2 (TMEFF2) is a transmembrane protein in the tomoregulin family. Little research has been performed to determine whether TMEFF2 methylation is a prognostic marker in adult diffuse gliomas.

**Methods:**

In this study, we investigated TMEFF2 expression in surgical glioma tissue samples. In addition, we conducted bisulfite amplicon sequencing (BSAS) and methylation-specific PCR (MSP) to evaluate TMEFF2 methylation in glioblastoma (GBM) cells. Subsequently, we investigated the biological function of TMEFF2 in GBM cells. Moreover, we explored the prognostic significance of TMEFF2 in gliomas by analysing a cohort dataset from TCGA.

**Results:**

Immunohistochemistry analysis of 75 paired glioma tumour and peritumoural tissues demonstrated that glioma tumour tissues expressed lower TMEFF2 levels than peritumoural tissues (P < 0.001). TMEFF2 promoter methylation levels were increased in glioblastoma cells compared with SVG p12 cells (P < 0.001). Inhibition of methylation reduced TMEFF2 methylation and increased its expression in LN229 and T98G cells (P < 0.05). Knockdown of TMEFF2 expression significantly promoted the proliferation of U87MG cells and primary GBM cells (P < 0.05). TMEFF2 methylation is negatively associated with IDH1, ATRX and TP53 mutations, and the subtype of glioma harbouring combined IDH1/ATRX/TP53 mutations was associated with low TMEFF2 methylation levels. Survival analysis confirmed that low TMEFF2 methylation levels are associated with good prognosis in glioma patients.

**Conclusions:**

Our results suggest that TMEFF2 DNA methylation might be associated with glioma tumour progression and could serve as a valuable prognostic marker for adult diffuse gliomas.

## Introduction

Diffuse gliomas account for approximately 80 % of malignant tumours originating in the central nervous system (CNS) in adults. Despite the development of standard treatments and other emerging treatments for adult diffuse gliomas, the outcomes of glioma patients remain relatively poor  [1, 2]. Inter- and intratumoural heterogeneity may contribute to the different outcomes of glioma patients [3, 4]. Based on these current issues, the updated 2016 World Health Organization (WHO) classification of tumours of the CNS emphasized the role of molecular markers, such as IDH1/2, EGFR, TP53, and MGMT, in the diagnosis and prognosis prediction of adult diffuse gliomas [2].

The isocitrate dehydrogenase (IDH) gene is mutated in > 70 % of diffuse lower-grade gliomas and in some glioblastomas [5, 6]. The mutant IDH protein produces the oncometabolite D-2-hydroxyglutarate (2HG), affecting epigenetic regulation, especially DNA methylation, of the genome of glioma cells [7, 8]. Despite its role in tumour initiation, mutant IDH is a hallmark of favour prognosis in glioma patients [9–11]. However, the outcomes for glioma patients with IDH mutations are also remarkably different [12]. Research on new markers would help us understand molecular events during adult diffuse glioma progression and provide better guidance for patient prognosis and treatment.

TMEFF2 (transmembrane protein with EGF-like and two follistatin-like domains 2, also known as HPP1 or TPEF) encodes a transmembrane protein of the tomoregulin family [13]. TMEFF2 is downregulated by promoter hypermethylation in several neoplastic diseases, such as colon cancer, oesophageal cancer, gastric cancer, and prostate cancer [14–18]. Interestingly, TMEFF2 can act as a promotor as well as suppressor during tumour progression, depending on alternative splicing and ectodomain shedding [19–22]. Although TMEFF2 promoter methylation levels vary in different adult glioblastoma subtypes in The Cancer Genome Atlas (TCGA) database [23], its expression and promoter methylation in glioma cells remain unconfirmed. Moreover, little is known about the correlation between TMEFF2 and the prognosis of adult diffuse gliomas.

In this study, we attempted to investigate TMEFF2 expression and promoter methylation in primary glioma tissue samples and in vitro cultured glioblastoma cells. We conducted TCGA database mining to evaluate the role of TMEFF2 promoter methylation as a potential prognostic marker in adult diffuse gliomas.

## Materials and methods

### Patients and tissue samples

Patients enrolled in this study were independently diagnosed with primary glioma by two pathologists in a double-blinded manner according to the criteria of the 2016 WHO classification. They had undergone routine surgery at the Department of Neurosurgery of Nanfang Hospital (Guangzhou City, Guangdong Province, China) between 2013 and 2019 without radiotherapy or chemotherapy prior to surgery. Brain tissues beyond MRI indicated peritumor edema area were collected as peritumor tissues. Temporal tissue samples from 3 epilepsy patients undergoing surgical treatment were used as normal brain tissues. This study was permitted by the Ethics Committee of Southern Medical University, and informed consent was obtained from each of the enrolled patients.

### Cell lines and culture

Human glioma cell lines U87MG, T98G, LN229, and SVG p12 were purchased from American Type Culture Collection (ATCC: Rockville, MD, USA). The primary human glioblastoma cell line NFHDCD was derived and cultured from a patient pathologically diagnosed with GBM (Male, Aged 60, GBM, WHO IV, IDH+, MGMT+, TP53+, Ki67+, EGFR+) who underwent routine surgery at the Department of Neurosurgery of Nanfang Hospital (Guangzhou City, Guangdong Province, China) [24]. Cells were maintained in Dulbecco’s modified Eagle’s medium (DMEM glucose 4.5 g/L; Biological Industries) with 10 % fetal bovine serum (FBS; Biological Industries) at 37 °C in a humidified incubator with 5 % CO_2_.

### Immunohistochemistry (IHC)

Seventy-five paired human glioma tumor and peritumor tissues were used for immunohistochemistry experiments to study altered TMEFF2 protein expression using the two-step plus poly-horseradish peroxidase (HRP) method. The clinical information of the studied specimens was shown in Additional file 1: Table S1. Briefly, 4 µm sections were mounted on amino propyl ethoxysilane (APES) slides. The slides were deparaffinized, rehydrated, immersed in 10 mM sodium citrate buffer (pH 6.0), pretreated in a microwave oven for 20 min, and then rinsed for 15 min with phosphate-buffered saline (PBS). Endogenous peroxidase was quenched by incubation of the sections in 0.3 % hydrogen peroxide for 30 min at room temperature. Nonspecific binding was blocked by incubation with nonimmune serum (1 % bovine serum albumin for 15 min at room temperature). The sections were incubated overnight with two polyclonal antibodies against TMEFF2 (rabbit anti-TMEFF2, ab50002, Abcam, Cambridge, UK; and rabbit anti-TMEFF2, 11928-1-AP, Proteintech, Rosemont, IL, USA) at a dilution of 1:1000. The next day, the slides were stained with a two-step plus Poly-HRP Anti-Rabbit IgG Detection System (PV-6001; ZSGB-Bio, Beijing, China) to detect TMEFF2. After visualization of the reaction with 3,3′-diaminobenzidine, the slides were counterstained with hematoxylin and mounted with synthetic medium. Gastric cancer tissue was used as a positive control, and PBS replaced the anti-TMEFF2 primary antibody to provide a negative control condition.

### RNA isolation and qRT-PCR

Twenty-three paired human glioma tumor and peritumor tissues were used in qRT-PCR experiments to detect TMEFF2 mRNA expression. The clinical information of the studied specimens was shown in Additional file 1: Table S2. RNA isolation and qRT-PCR were carried out as previously described [25]. Total RNA was isolated from twenty-three paired glioma tumor and peritumor tissues as well as LN229, T98G, U87MG, and NFHDCD cells using TRIzol (Invitrogen), and RNA samples (600 ng per sample) were used to generate cDNA using a PrimeScript™ RT reagent Kit with gDNA Eraser (Takara, Cat# RR047A) according to the manufacturers’ instructions. The obtained cDNA samples were used as templates for qPCR amplifications using TB Green® Premix Ex Taq™ II (Tli RNaseH Plus) (Takara, Cat# RR820A). GAPDH was used as a corresponding internal control. All mRNA levels were quantified by the 2-ΔΔCT method. Each reaction was performed in triplicate. The primer sequences of TMEFF2 were as follows: forward 5’-GCTGCTTTCCCTACCTCCTT-3’; reverse 5’-AGCCACACACAGGCACATAG-3’. The primer sequences of GAPDH were as follows: forward 5’-TGACTTCAACAGCGACACCCA-3’; reverse 5’-CACCCTGTTGCTGTAGCCAAA-3’. The primer sequences of DNMT1 were as follows: forward 5’-GATCTCCTACAACGGGGAGC-3’; reverse 5’-AGCCACCAATGCACTCATGT-3’.

### DNA bisulfite conversion and methylation analysis

A DNA extraction kit (Takara, Cat# 9765) was used to isolate DNA from GBM and SVG p12 cells according to the manufacturer’s instructions. Sodium bisulfite conversion of 600 ng of extracted DNA was performed using a DNA Bisulfite Conversion Kit (Tiangen, Cat# DP215-02). Bisulfite conversion was followed by bisulfite amplicon sequencing (BSAS) or methylation-specific PCR (MSP). Bisulfite amplicon sequencing (BSAS) was performed within CpG sites of the promoter region of TMEFF2. The four primers that were used to amplify TMEFF2 are shown in Additional file 1: Table S3. Methylation data were then analyzed, and the average methylation levels at all sites were calculated using MethylKIT software (Access Date 2019/09/26). The methylation level of each CpG site is defined as the ratio of the number of methylated reads to the combination of methylated and unmethylated reads (values between 0 and 1). For methylation-specific PCR, an EpiScope MSP Kit (Takara, Cat# RR100A) was used, and qRT-PCR was performed. The primers specific for TMEFF2 in MSP are shown in Additional file 1: Table S3. For MSP of clinical glioma samples, tumor tissues from forty-three patients who had undergone whole exome sequencing in the clinic, which determined the mutation status of the IDH1, ATRX and TP53 genes, were used. The clinical information of the studied specimens was shown in Additional file 1: Table S4. Temporal tissue samples from 3 epilepsy patients undergoing surgical treatment were used as normal controls.

### 5-Aza-2-deoxycytidine decitabine (DAC) treatment

DAC was purchased from Selleck Chemical Co. (Selleck, Cat# S1200) and was dissolved in dimethyl sulfoxide (DMSO) (Sigma, Cat# D2650). The stock solution was diluted with PBS to an original concentration of 10 mM and stored at – 20℃. Further working solutions were added to the cell culture medium immediately before use. Appropriate DMSO controls were implemented. Cells were treated with DAC at a concentration of 5 µM for 96 h, after which total RNA or DNA was extracted from the cells.

### Transient knockdown of DNMT1 and TMEFF2 in GBM cells

Cells were transfected with chemosynthetized siRNAs purchased from Kidan Biotechnology Co. (Guangzhou, China) using Lipofectamine 2000 reagent (Invitrogen, Cat# 11,668) for 8 h according to the manufacturer’s protocol. The sequences of the siRNAs are shown in Additional file 1: Table S3.

### Cell proliferation assay

Cell proliferation was measured by Cell Counting Kit-8 (CCK8) assays and EdU assays.

For CCK8 assays, cells were seeded in 96-well plates at a density of 2000 cells/well and then were incubated for 12 h to allow cell attachment. Then, 10 µL of CCK-8 solution (Bimake, Cat# B34304) was added to each well on days 1, 2, 3, 4, 5 and 6, which was followed by another 2 h incubation. The optical density was then measured at 450 nm using a microplate reader.

For EdU assays, cells were plated at a density of 20,000/dish in confocal dishes. After 24 h of incubation, cells were treated with EdU reagent (RiboBio, Cat# C10310-1) for 2 h according to the manufacturer’s instructions and then were fixed with 4 % paraformaldehyde. One hundred microliters of 1X Apollo^®^567 staining reaction solution and Hoechst 33,342 (RiboBio, Cat# C10310-1) was added to each dish and then was incubated for 30 min at room temperature on a decolorization shaker. Cells were then visualized using a BX63 automatic intelligent fluorescence microscope (Olympus, Tokyo, Japan).

### Database and data analysis

Validation cohort data was collected from the GBM and LGG projects of The Cancer Genome Atlas (TCGA). DNA Methylation (Illumina 450K), Mutations and Clinical Data sets were downloaded from the cBioPortal database [26], and RNA HiSeq V2 RSEM data were downloaded from the GDC Data Portal [27]. RNA HiSeq V2 RSEM data were transformed from FPKM into TPM data before merging into the matrix. 

### Statistical and survival analysis

R software version 3.5.0 was used to assess the relationship between the TMEFF2 methylation level and mRNA expression through the use of Spearman’s correlation analysis. Kruskal-Wallis tests were used to analyze TMEFF2 methylation or mRNA expression in different cells as well as in different tumor grades. The Wilcox test was used to analyze the correlation between TMEFF2 methylation or mRNA expression and gene mutations. Kaplan-Meier analysis was used to assess the association of TMEFF2 methylation or TMEFF2 mRNA expression with patient prognosis. A scanning model found that β value = 0.1 was the best cutoff for TMEFF2 methylation and that TPM = 6.07 was optimal for assessing TMEFF2 mRNA expression in Kaplan-Meier analysis of the TCGA cohort. Differences between survival rates were analyzed using a log-rank test, and survival curves were plotted using R software. Differences in TMEFF2 methylation and expression in different treatment groups were analyzed using Student’s t test, and CCK8 results were analyzed using variance analysis of two-factor repeated measures. Statistical analyses were carried out using SPSS statistical software (version 20.0, SPSS, Inc., Chicago, IL, USA), and the significance level was assigned at P < 0.05.

## Results

### TMEFF2 is hypermethylated, and its expression is reduced in gliomas

**Fig. 1:**
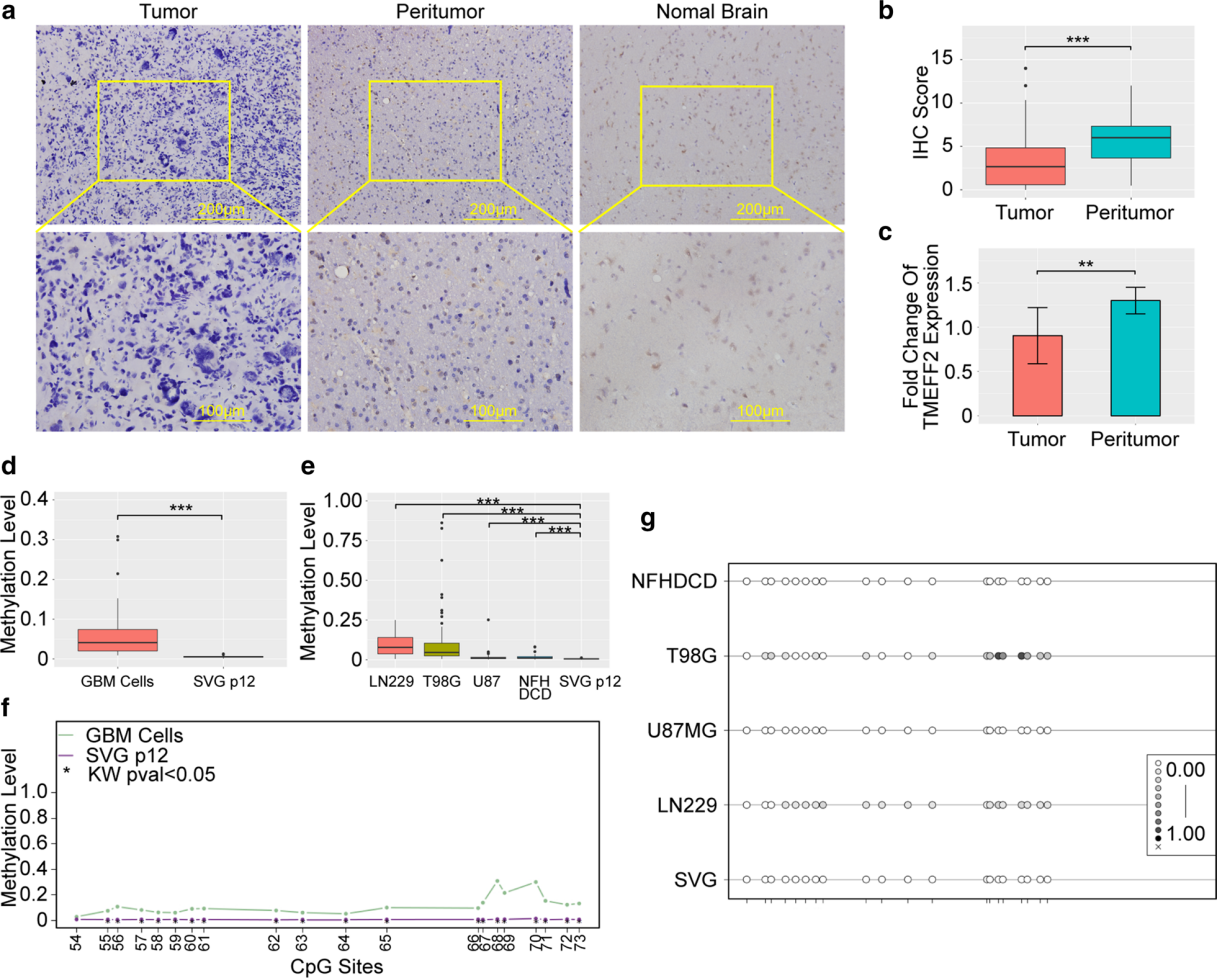
TMEFF2 expression and methylation in glioma tissues and glioblastoma cells. **a** Representative images of TMEFF2 immunohistochemical staining of paired tumor/peritumor tissues from one glioma patient and tissue from one epilepsy patient. **b** Boxplot of the TMEFF2 immunohistochemistry staining score in tumor and peritumor tissues (n = 75). **c** Bar gragh of the RT-PCR of TMEFF2 expression in paired tumor/peritumor glioma patient tissues (n = 23). Error bars represent the SD of tissues. **d** Boxplot of the total methylation levels of BSAS in GBM cells and SVG p12 cells. **e** Boxplot of the total methylation levels of BSAS in each GBM cell and SVG p12 cell sample. **f** Line plot of CpG sites of the fourth CpG island of the TMEFF2 promoter in GBM cells and SVG p12 cells. **g** Methylation plot of the fourth CpG island of the TMEFF2 promoter in GBM cells and SVG p12 cells. CpG sites are presented at each point on each line. **P < 0.01, and ***P < 0.001

After evaluating immunostained paired tumour and peritumoural specimens from 75 glioma patients and normal brain specimens from 5 epilepsy patients, we observed a relatively low level of TMEFF2 protein expression in 70.67 % (53/75) of all tumour specimens. TMEFF2 expression in tumour specimens was lower than that in peritumoural specimens (P < 0.001) (Fig. 1a, b). The immunohistochemistry staining results were consistent when using another rabbit anti-TMEFF2 antibody (data not shown). RT-PCR of 23 paired glioma samples showed that TMEFF2 mRNA expression levels were lower in tumour tissues than in peritumour tissues (P < 0.01) (Fig. 1c). To identify changes in methylation of the TMEFF2 promoter in glioma, we performed bisulfite amplicon sequencing (BSAS) in 1 patient-derived primary glioblastoma cell line (NFHDCD) and 3 glioblastoma cell lines (T98G, LN229, and U87MG), and we also analysed a normal astroglial cell line (SVG p12). Using four pairs of different primers, we amplified the DNA sequences of four CpG islands in the TMEFF2 promoter and detected the methylation levels of all CpG sites involved in these islands to generate a landscape of TMEFF2 promoter methylation in GBM and SVG p12 cells. All glioblastoma cells exhibited higher average TMEFF2 promoter methylation levels than did SVG p12 cells (P < 0.001) (Fig. 1d, e). To further interrogate the methylation sites of TMEFF2 in glioma, we identified 49 differentially methylated cytosines (DMCs) in a total of 73 CpG sites in the TMEFF2 promoter that were hypermethylated in glioblastoma cells compared to SVG p12 cells (Additional file 1: Table S5, Fig. 1f, g, Additional file 1: Fig S1a–f).

### TMEFF2 expression is negatively correlated with its promoter methylation level in glioblastoma cells

**Fig. 2:**
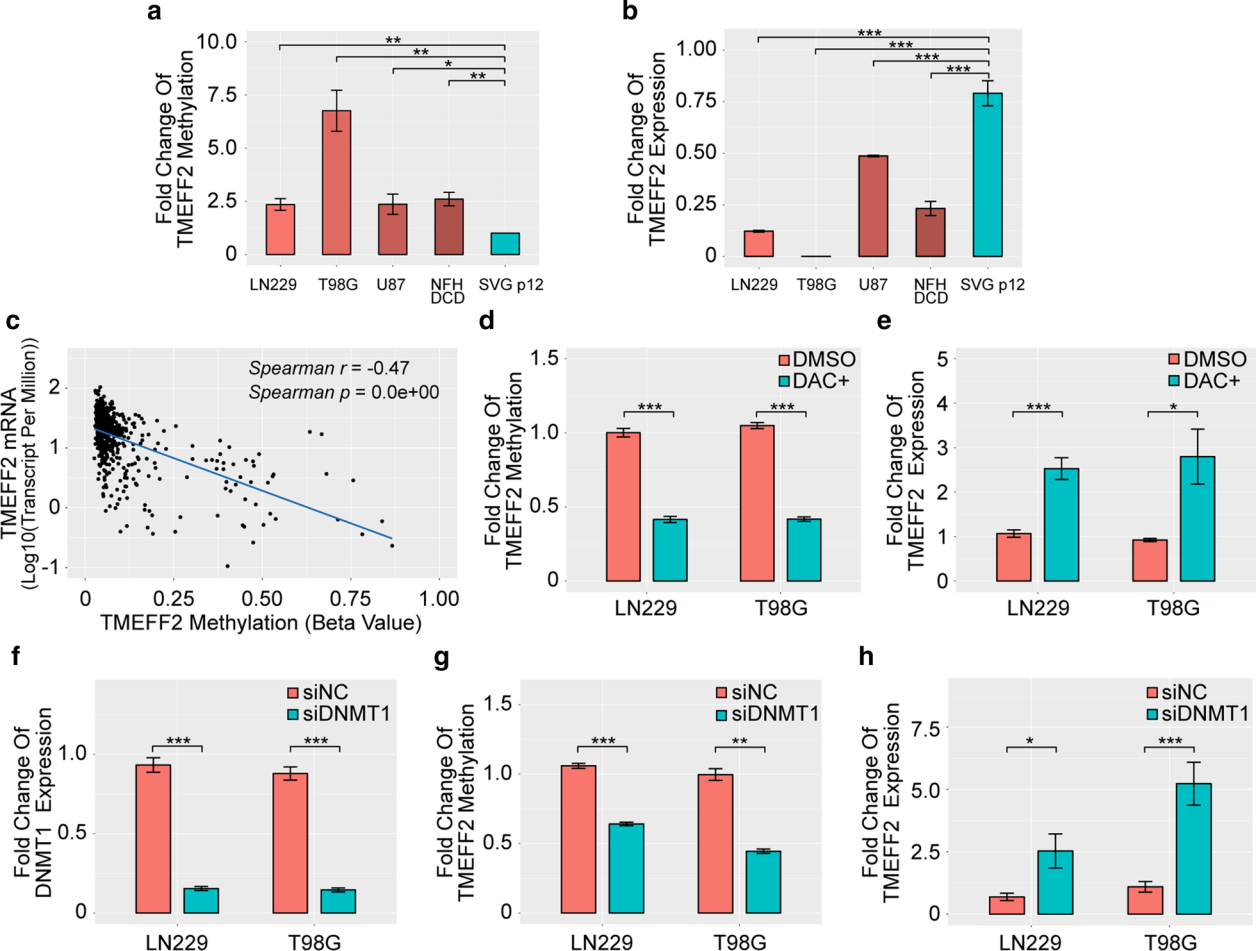
Inhibition of methylation upregulates TMEFF2 mRNA expression in glioblastoma cells. **a** Bar graghBar gragh of the MSP analysis of GBM cells and SVG p12 cells. Error bars represent the SD of repeats of each cell. **b** Bar gragh of the RT-PCR analysis of GBM cells and SVG p12 cells. Error bars represent the SD of repeats of each cell. **c** The correlation between TMEFF2 methylation and mRNA expression in primary gliomas (n = 565). **d** Bar gragh of the MSP analysis of TMEFF2 in LN229 and T98G cells following treatment with DAC (5 µM) for 96 h. Error bars represent the SD of repeats of each cell. **e** Bar gragh of the RT-PCR analysis of TMEFF2 in LN229 and T98G cells following treatment with DAC (5 µM) for 96 h. Error bars represent the SD of repeats of each cell. **f** Bar gragh of the RT-PCR analysis of DNMT1 in LN229 and T98G cells treated with DNMT1 siRNA. Error bars represent the SD of repeats of each cell. **g** Bar gragh of the MSP analysis of TMEFF2 in LN229 and T98G cells following treatment with DNMT1 siRNA. Error bars represent the SD of repeats of each cell. **h** Bar gragh of the RT-PCR analysis of TMEFF2 in LN229 and T98G cells treated with DNMT1 siRNA. Error bars represent the SD of repeats of each cell. *P < 0.05, **P < 0.01, and ***P < 0.001

Methylation-specific PCR (MSP) verified TMEFF2 promoter hypermethylation in glioblastoma cells (Fig. 2a). Conversely, RT-PCR demonstrated that the TMEFF2 mRNA expression was lower in glioblastoma cells than in the other cells tested (Fig. 2b). To confirm the correlation between TMEFF2 promoter methylation and expression in gliomas, we queried a dataset from TCGA and observed a significant negative correlation (Spearman’s r = – 0.47, P < 0.001) between the TMEFF2 promoter methylation level and mRNA expression in glioma (Fig. 2c). Treatment with the demethylating agent decitabine (DAC) decreased TMEFF2 promoter methylation to 41.48 % of untreated levels (P < 0.001) in LN229 cells and 39.85 % of untreated levels (P < 0.001) in T98G cells (Fig. 2d). Furthermore, treatment with DAC caused a 2.97-fold (P < 0.001) and 3.03-fold (P < 0.05) increase in TMEFF2 mRNA in LN229 and T98G cells, respectively (Fig. 2e). Furthermore, we used siRNAs to knock down the expression of the human DNA methyltransferase DNMT1 in LN229 and T98G cells. The efficiency of DNMT1 interference in both cell lines was verified by RT-PCR (Fig. 2f). Knocking down DNMT1 inhibited TMEFF2 promoter methylation to 60.51 % (P < 0.01) and 44.65 % (P < 0.001) and upregulated TMEFF2 mRNA expression by 3.677-fold (P < 0.001) and 4.76-fold (P < 0.05), in the LN229 and T98G cell lines, respectively (Fig. 2g, h).

### TMEFF2 is potentially involved in the regulation of glioblastoma cells proliferation

**Fig. 3:**
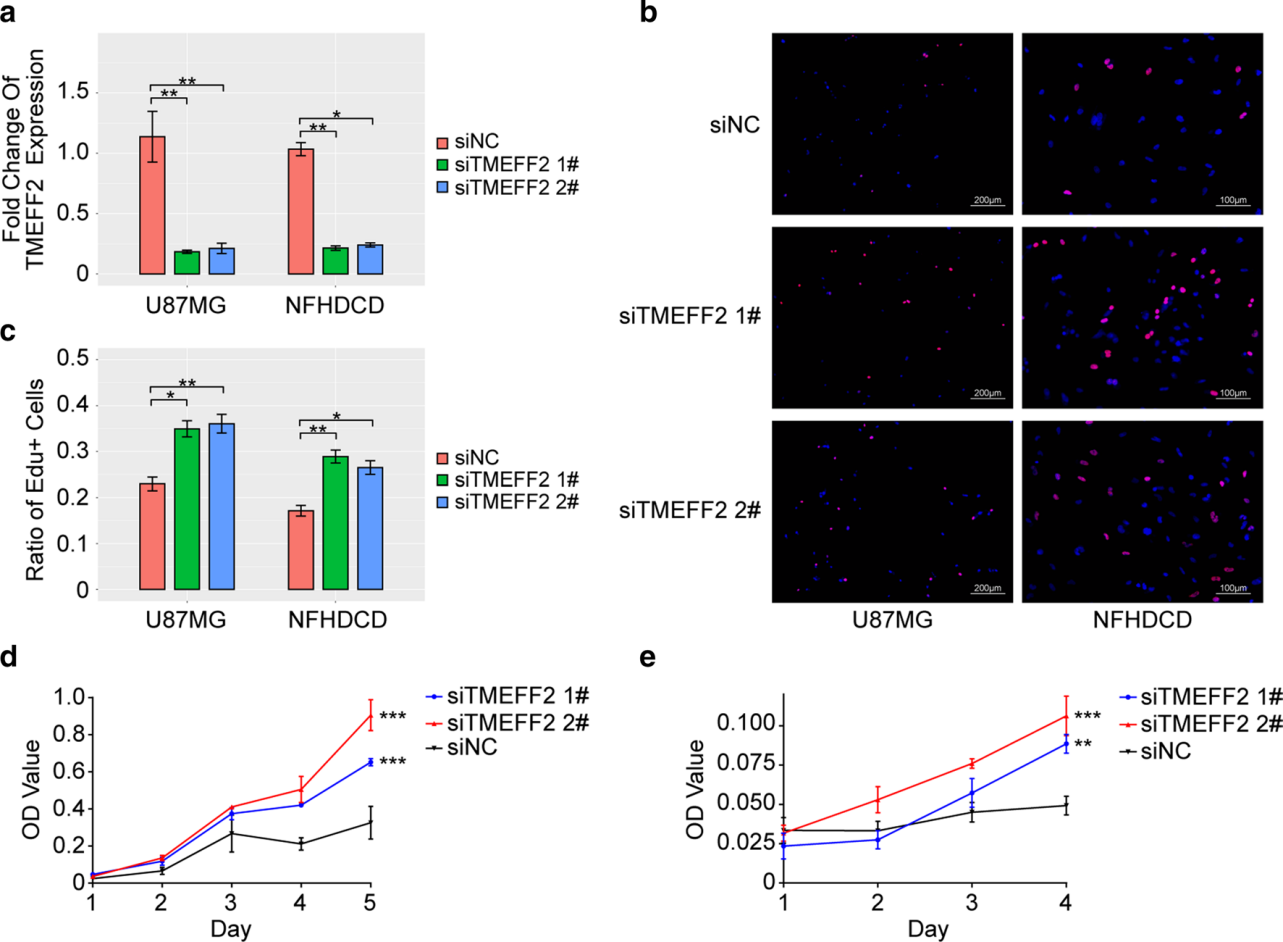
Knockdown of TMEFF2 in U87 and NFHDCD cells enhanced GBM cell proliferation. **a** Bar gragh of the RT-PCR of TMEFF2 mRNA expression in U87MG and NFHDCD cells. Error bars represent the SD of repeats of each cell. **b** and **c** EdU assay showing different cell proliferation rates in siTMEFF2- and siNC-treated U87MG and NFHDCD cells. Error bars represent the SD of repeats of each cell. **d** and **e** CCK8 assay showing different cell proliferation rates in siTMEFF2- and siNC-treated U87MG and NFHDCD cells, respectively. *P < 0.05, **P < 0.01, and ***P < 0.001

To verify the biological function of the TMEFF2 protein in glioblastoma cells, we examined the effects of TMEFF2 knockdown by two distinct siRNAs on the glioblastoma cell line U87MG and the primary glioblastoma cell line NFHDCD. Compared with a nontargeting control siRNA (siNC), treatment with either TMEFF2 siRNA significantly reduced TMEFF2 expression in both cell lines (Fig. 3a). Knockdown of TMEFF2 in both U87MG and NFHDCD glioblastoma cells significantly increased the ratio of EdU + cells as demonstrated by EdU tests (Fig. 3b, c), and knockdown promoted cell proliferation, as demonstrated by CCK8 tests (Fig. 3d, e). Thus, TMEFF2 might act as an inhibitor of the proliferation of adult diffuse glioma.

### TMEFF2 promoter methylation is potentially negatively correlated with the IDH1+/ATRX+/TP53 + glioma subtype

**Fig. 4:**
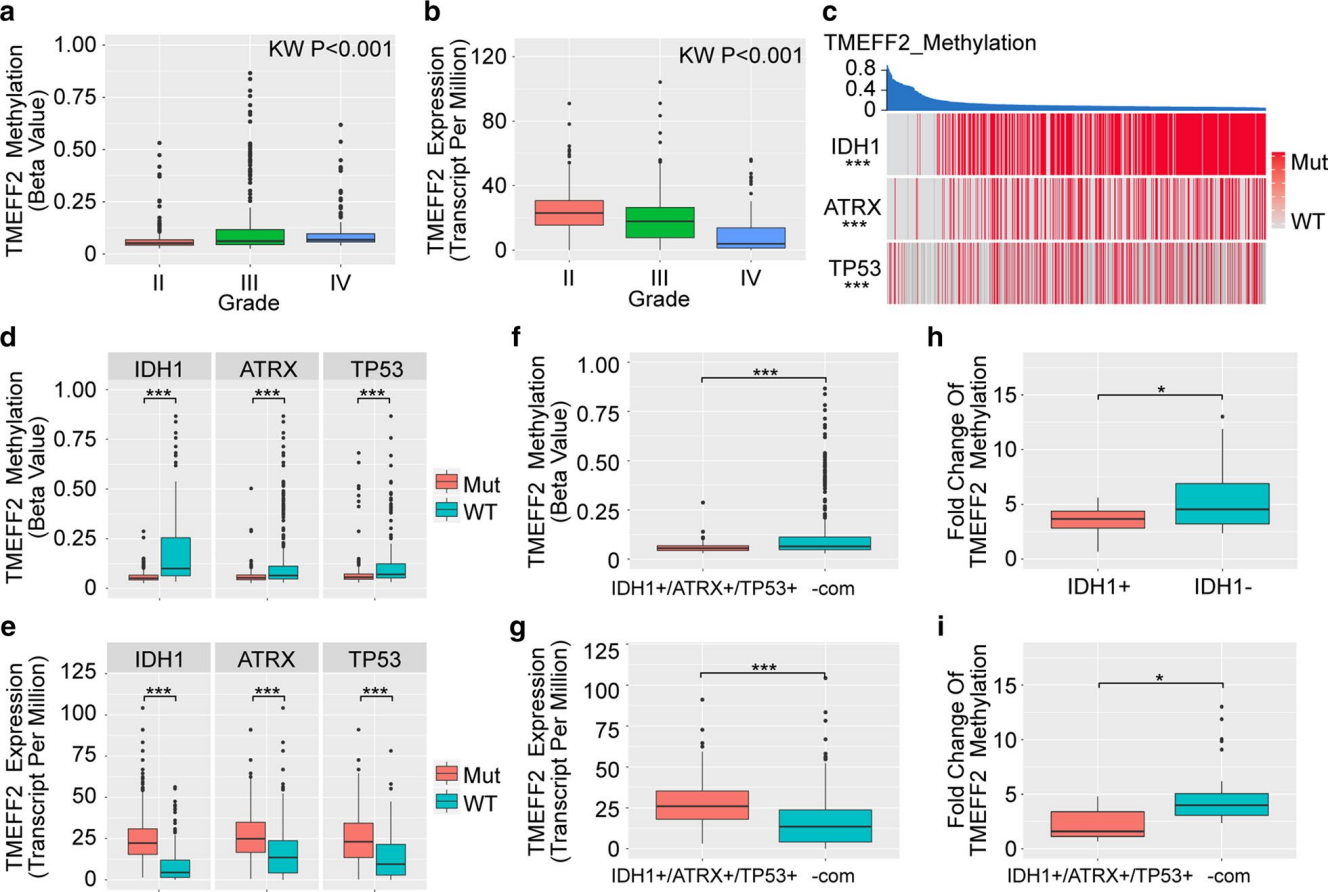
TMEFF2 methylation and mRNA expression levels in the TCGA dataset. **a** Boxplot of the TMEFF2 methylation level in different grades of primary gliomas (n = 1122). Kruskal-Wallis P < 0.001. **b** Boxplot of the TMEFF2 mRNA expression in different grades of primary gliomas (n = 1122). Kruskal-Wallis P < 0.001. **c** Negative association of TMEFF2 methylation with IDH1, ATRX, and TP53 mutations in primary gliomas. **d** Boxplot of the TMEFF2 methylation level in IDH1, ATRX and TP53 mutant/wild-type primary gliomas. **e** Boxplot of the TMEFF2 mRNA expression levels in IDH1, ATRX and TP53 mutant/wild-type primary gliomas. **f** Boxplot for TMEFF2 methylation level in IDH1/ATRX/TP53 combined mutant and non-combined mutant primary gliomas (n = 573). **g** Boxplot of the TMEFF2 mRNA expression in IDH1/ATRX/TP53 combined mutant and non-combined mutant primary gliomas (n = 588). **h** Boxplot for TMEFF2 methylation level in IDH1 mutant and IDH1 wild-type primary glioma patients’ tumor tissues. **i** Boxplot for TMEFF2 methylation level in IDH1/ATRX/TP53 combined mutant and non-combined mutant primary glioma patients’ tumor tissues. *P < 0.05, and ***P < 0.001

To further explore the correlation between TMEFF2 methylation and adult diffuse glioma, we combined the Brain Low Grade Glioma and Glioblastoma Multiforme datasets from TCGA and generated a pan-glioma cohort with 1122 primary glioma samples (Additional file 1: Table S6). In our cohort, TMEFF2 promoter methylation was found to increase with glioma tumour grade (Fig. 4a); inversely, its mRNA expression was reduced from low- to high-grade glioma (Fig. 4b). Given that adult diffuse gliomas are highly heterogeneous, we hypothesized that TMEFF2 promoter methylation was correlated with one of the glioma subtypes. To test this hypothesis, we filtered gene mutations associated with TMEFF2 promoter methylation and found that TMEFF2 promoter methylation was negatively correlated with IDH1 mutation, ATRX mutation and TP53 mutation (Fig. 4c). In addition, in either IDH1 or ATRX or TP53 mutant samples, TMEFF2 was hypomethylated, and its mRNA expression was upregulated (Fig. 4d, e). Next, we divided the samples in our cohort into the IDH1, ATRX and TP53 combined mutant group (IDH1+/ATRX+/TP53+) and the -combined mutant group (-com, IDH1 wild-type and IDH1 mutant but ATRX or TP53 wild-type). Compared to the -com group, IDH1+/ATRX+/TP53 + gliomas exhibited lower TMEFF2 promoter methylation (P < 0.001) and higher TMEFF2 mRNA expression (P < 0.001) (Fig. 4f, g). Furthermore, we verified these results in clinical glioma tissues. IDH1 mutant glioma tissues (n = 23) harboured lower TMEFF2 methylation levels than IDH1 wild-type glioma tissues (n = 20) (P < 0.05) (Fig. 4h). IDH1+/ATRX+/TP53 + glioma tissues (n = 6) exhibited lower TMEFF2 methylation levels than non-combined mutant glioma tissues (n = 37) (P < 0.05) (Fig. 4i).

### TMEFF2 promoter hypomethylation might be an indicator of better OS in patients with IDH1 + glioma

**Fig. 5:**
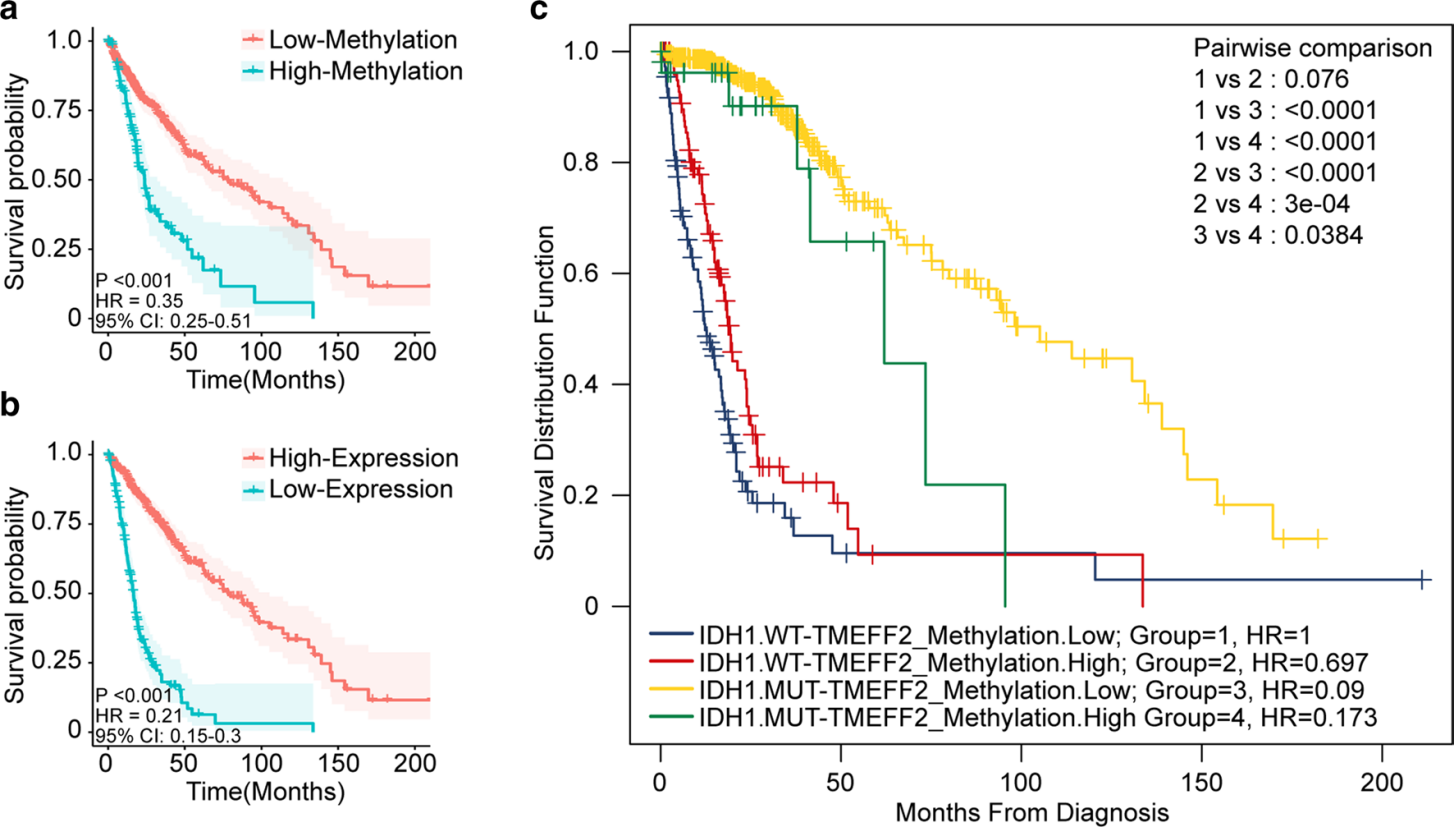
Prognostic value of TMEFF2 methylation in primary gliomas in TCGA dataset. **a** Kaplan-Meier curve for OS (month) in patients with low levels of TMEFF2 methylation (n = 519) versus high levels of TMEFF2 methylation (n = 137) in primary glioma. **b** Kaplan-Meier curve for OS (month) in patients with low levels of TMEFF2 mRNA expression (n = 164) versus high levels of mRNA expression (n = 501) in primary glioma. **c** OS (month) for four subgroups of primary glioma patients stratified by combinations of both factors: patients with mutant IDH1 and low levels of TMEFF2 methylation (IDH1.MUT-TMEFF2_Methylation.Low), patients with mutant IDH1 and high levels of TMEFF2 methylation (IDH1.MUT-TMEFF2_Methylation.High), patients with wild-type IDH1 and low levels of TMEFF2 methylation (IDH1.WT-TMEFF2_Methylation.Low), and patients with wild-type IDH1 and high levels of TMEFF2 methylation (IDH1.WT-TMEFF2_Methylation.High)

To determine the correlation between TMEFF2 promoter methylation and overall survival (OS) in glioma patients, we initially performed Kaplan-Meier survival curves with a log-rank test based on the best separation model. The results showed that patients whose primary tumours showed low TMEFF2 methylation achieved longer survival and a better prognosis than patients whose primary tumours exhibited high TMEFF2 methylation (HR = 0.35, P < 0.001; Fig. 5a). Additionally, survival analysis of TMEFF2 expression in primary gliomas implicated high expression of TMEFF2 in better overall survival than low expression (HR = 0.21, P < 0.001; Fig. 5b).

To further understand the prognostic value of TMEFF2 promoter methylation, we performed pairwise comparisons of the 4 cohorts grouped by the various combinations of IDH1 mutation (MUT/WT) and TMEFF2 promoter methylation (Low/High). We observed that samples with IDH1.MUT-TMEFF2_Methylation.Low showed the best outcome with the lowest HR of OS (HR = 0.09) (Fig. 5c). Comparing IDH1.MUT-TMEFF2_Methylation.Low with IDH1.MUT-TMEFF2_Methylation.High alongside IDH1.WT-TMEFF2_Methylation.Low and IDH1.WT-TMEFF2_Methylation.High, we found that among 427 IDH1 mutant samples, patients with low TMEFF2 promoter methylation (n = 400/427) exhibited better outcomes than patients with high TMEFF2 promoter methylation (n = 27/427), with an OS of 105.12 months versus 61.96 months (P = 0.038).

## Discussion

TMEFF2 is a transmembrane protein in the tomoregulin family that is hypermethylated and underexpressed in various tumour types. In gastric cancer, TMEFF2 deregulation may play an important role in the progression of gastric carcinogenesis [21]. However, in prostate cancer, the extracellular domain of TMEFF2 is shed from the cell membrane and promotes cell proliferation by combining with the ErbB1 receptor [22]. In the current study, we assessed TMEFF2 expression and promoter methylation and explored its clinical implications in adult diffuse glioma.

TMEFF2 is highly expressed in human brain tissues [17]. In our current study, we confirmed that compared to that in healthy tissue, TMEFF2 expression is significantly decreased in glioma tumour tissues and GBM cells. In addition, using data of TCGA GBM and LGG cohorts, we ascertained that TMEFF2 expression is progressively downregulated during the progression from Grade II to Grade IV glioma. Our data are also consistent with previous reports that TMEFF2 expression is downregulated and negatively correlated with tumour histologic grade in gastric cancer and colon cancer [18, 28].

High TMEFF2 expression levels are associated with both growth-promoting and growth-suppressing functions in various studies in multiple cancers. In most studies, TMEFF2 has been found to suppress growth [19–21], whereas Nazim Ali and Vera Knauper proposed that shedding of the soluble TMEFF2 ectodomain would induce proliferation by inducing ERK1/2 phosphorylation in an ErbB1-dependent manner in prostate cancer cells [22]. In our study, cell biological function assays showed increased proliferation in TMEFF2 knockdown GBM cells, indicating that TMEFF2 inhibited GBM cell proliferation. These data suggest that TMEFF2 may play important roles in suppressing the growth of adult diffuse glioma. Downregulation of TMEFF2 may be related to glioma tumour progression.

We performed bisulfite amplicon sequencing (BSAS) in GBM cells compared with SVG p12 cells and provided the first identification of hypermethylated CG sites in the TMEFF2 promoter of GBM cells. Our MSP work also demonstrated TMEFF2 hypermethylation in GBM cells. Moreover, we confirmed the negative correlation between TMEFF2 methylation and mRNA expression. Taken together, these results indicate that low TMEFF2 expression in gliomas may be due to methylation regulation of its promoter. It was demonstrated that histone deacetylases as well as c-Myc, STAT1 and STAT3 may contribute independently to the transcriptional suppression of TMEFF2 in colon cancer, prostate cancer and gastric cancer [29–32]. However, more detailed studies need to be performed to better understand the regulatory mechanism of TMEFF2 promoter methylation and transcription in gliomas.

However, we failed to clarify TMEFF2 expression in glioma tumour tissues or cells by Western blot (WB) using commercial antibodies (data not shown). We noticed that O-methylguanine-DNA methyltransferase (MGMT) is an important biomarker for the chemosensitivity of gliomas, and methylation detection (not IHC) is more precise for the clinical testing of MGMT in glioma patients [1, 33, 34]. Andreas Herbst et al. and Su Man Lee et al. detected methylated free-circulating DNA (ctDNA) for TMEFF2 in the blood of metastatic colorectal cancers and non-small cell lung cancer, respectively [35, 36]. It has also been reported that sequencing ctDNA in cerebrospinal fluid (CSF) can provide a landscape of the tumour genome for glioma patients and is associated with disease outcome [37]. In this study, we successfully and stably detected TMEFF2 DNA methylation levels in GBM cells and in glioma patients’ tumour tissues. Thus, methylated TMEFF2 DNA could also be a used as a detectable indicator in glioma patients’ tumour specimens or cerebrospinal fluid in the future.

Mounting evidence has confirmed that mutated IDH1 is a hallmark of favourable patient outcomes in adult diffuse gliomas. In our previous research, we demonstrated that IDH1 mutation occurs frequently and predicts favourable prognosis in Chinese glioma patients [38]. Mutant IDH1 in gliomas causes broad epigenetic alterations, including DNA hypermethylation, and results in a subtype of glioma with a CpG island methylator phenotype (G-CIMP) [39, 40]. In our study, we observed a small subset (27/427) of IDH1 mutant gliomas with a high degree of TMEFF2 methylation that exhibited a poor prognosis compared to the large subset (400/427), which had less TMEFF2 methylation. Due to tumour heterogeneity among glioma patients, TMEFF2 methylation may be a biomarker of poor prognosis in IDH1 mutant glioma patients. Genome-wide methylation of G-CIMP gliomas shifts substantially in tumour recurrence [41]. Our data have not demonstrated whether TMEFF2 methylation is associated with malignant tumour transformation during tumour recurrence. More studies could be performed to clarify the role of TMEFF2 methylation in tumour recurrence.

Gliomas with mutant IDH and 1p/19q noncodeletion mostly harbour loss-of-function mutations in ATRX and gain of new function mutations in TP53 [2]. In low-grade gliomas, this subtype is mainly composed of astrocytoma and anaplastic astrocytoma and displays a favourable prognosis. In glioblastomas, mutated IDH is present in only < 10 % of all cases, but in those cases there is a better prognosis [10, 42]. The molecular profiles of glioblastoma with mutated IDH are similar to those of astrocytoma with mutated IDH, including frequent ATRX and TP53 mutations and MGMT hypermethylation. In our study, through TCGA database analysis and clinical specimen verification, we found that TMEFF2 promoter methylation is negatively correlated with IDH1, ATRX and TP53 mutations. The IDH1, ATRX and TP53 combined mutant (IDH1+/ATRX+/TP53+) samples in our glioma cohort presented lower levels of TMEFF2 promoter methylation and higher levels of TMEFF2 expression compared with other samples. Thus, low TMEFF2 methylation may be a new detectable molecular marker used to identify IDH1+/ATRX+/TP53 + gliomas.

## Conclusions

In this study, we assessed TMEFF2 promoter methylation and expression in glioma and highlighted the clinical significance of TMEFF2 methylation in glioma. The TMEFF2 methylation level may serve as a prognostic marker for adult diffuse gliomas. Low TMEFF2 methylation may be a new molecular marker used to identify IDH1+/ATRX+/TP53 + gliomas. Further molecular, cellular, and animal model studies should be performed to achieve a comprehensive understanding of the mechanism of TMEFF2 in carcinogenesis and tumour progression in adult diffuse gliomas.

## Supplementary Information

**Additional file 1.** Supplementary Metierial 1 (TableS1–S6 and Fig S1)

## Data Availability

Not applicable.
